# Prepatellar Morel-Lavallée Effusion Mimicking a Bursitis

**DOI:** 10.7759/cureus.59129

**Published:** 2024-04-27

**Authors:** Hamza Retal, Nicolas Cavez, Erika De Smet, Wiem Abid, Redouane Kadi

**Affiliations:** 1 Radiology, Helora University Hospital, Nivelles, BEL; 2 Radiology, Humani Univesity Hospitals of Charleroi-Chimay, Charleroi, BEL

**Keywords:** non-surgical orthopedics, mri, prepatellar bursitis, knee, morel-lavallée effusion

## Abstract

Morel-Lavallée lesions are traumatic abnormalities characterized by the accumulation of hemolymphatic fluid collection following disruption of subcutaneous fat tissue from the underlying deep fascia. Here, we present the case of a 63-year-old woman with a prepatellar Morel-Lavallée lesion, an unusual location for this pathology initially misdiagnosed as prepatellar bursitis.

An MRI was performed allowing the correction of the diagnosis, highlighting the essential role of imaging in confirming the diagnosis and ruling out differentials such as prepatellar bursitis or neoplastic origins. In our case, conservative treatment with compression alone was employed, since surgery is reserved for chronic or complicated cases.

Our experience underscores the utility of MRI in accurately delineating the anatomical extent and characteristics of prepatellar Morel-Lavallée effusion (PMLE). This imaging modality serves as a critical tool in guiding appropriate management strategies, ensuring timely and effective treatment for patients presenting with this lesion.

## Introduction

Morel-Lavallée lesions represent a unique clinical entity characterized by the accumulation of hemolymphatic fluid following traumatic disruption of subcutaneous fat tissue from the underlying deep fascia. Although these lesions typically manifest in areas such as the hip, thigh, and pelvis, occurrences in the prepatellar region are infrequent yet not uncommon, posing diagnostic challenges and considerations for therapy. A literature review by Vanhegan et al. identified 204 lesions from 29 articles, revealing a 15.7 % incidence of prepatellar Morel-Lavallée lesions [1]. Herein, we present a case report detailing the clinical presentation, imaging findings, and management approach for a prepatellar Morel-Lavallée lesion in a 63-year-old woman initially misdiagnosed as prepatellar bursitis.

Prepatellar Morel-Lavallée effusion (PMLE) is an uncommon condition often resulting from traumatic shearing or compressive forces, leading to the separation of subcutaneous fat from the deep fascia and subsequent fluid collection. Misinterpretation of PMLE as prepatellar bursitis is plausible, emphasizing the importance of accurate diagnosis through advanced imaging modalities such as magnetic resonance imaging (MRI). In our case, imaging played a pivotal role in confirming the diagnosis, highlighting its essential place in guiding appropriate management strategies.

While conservative measures remain the cornerstone of treatment for acute PMLE, chronic or complicated cases may necessitate surgical intervention. Understanding the clinical, radiological, and therapeutic aspects of PMLE is crucial for optimizing patient care and outcomes, particularly in scenarios where misdiagnosis may lead to suboptimal management strategies. Therefore, this case report underscores the significance of recognizing and effectively managing PMLEs, offering insights into diagnostic approaches, therapeutic considerations, and outcomes associated with this relatively uncommon presentation.

Through a comprehensive examination of this case and review of relevant literature, we aim to provide clinicians and radiologists with valuable insights into the diagnostic challenges, therapeutic options, and prognostic factors associated with prepatellar Morel-Lavallée lesions, ultimately contributing to enhanced patient care and improved clinical outcomes.

## Case presentation

**Figure 1:**
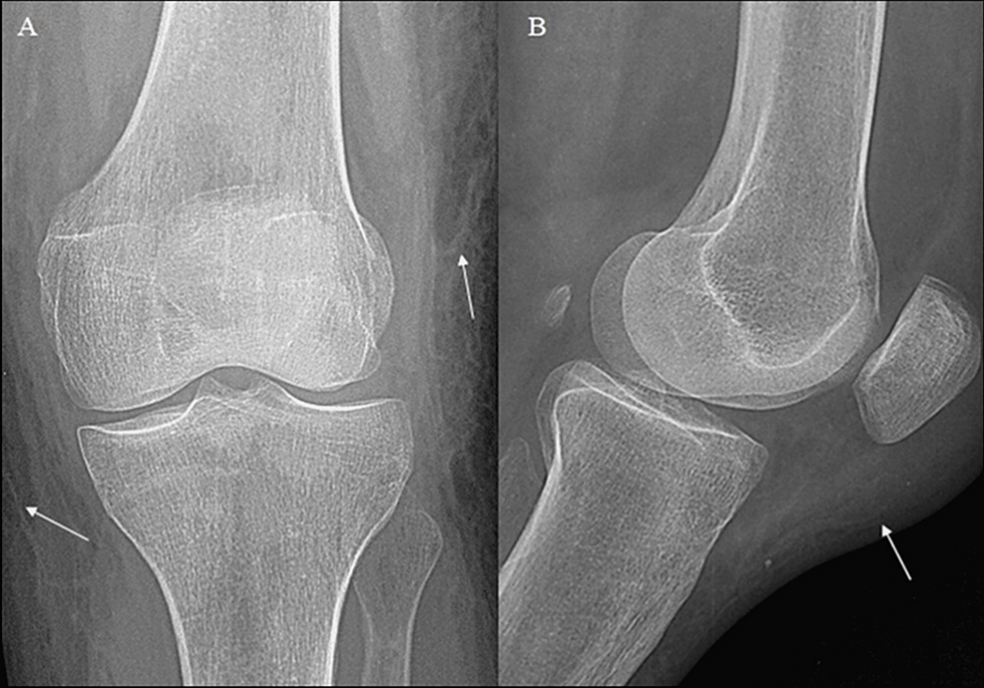
Antero-posterior (A) and lateral (B) plain radiography of the left knee depict a soft tissue swelling (arrows, A and B) predominant in the prepatellar aspect of the knee (arrow, B), with no evidence of bone or articular lesions, nor signs of intra-articular effusion.

The present case involves a 63-year-old female patient with a medical history notable for schizophrenia, who presented with left knee pain and swelling subsequent to repeated falls over a span of several weeks. Initial evaluation through lateral plain radiography of the left knee revealed a conspicuous prepatellar soft tissue swelling (Figure 1).

**Figure 2:**
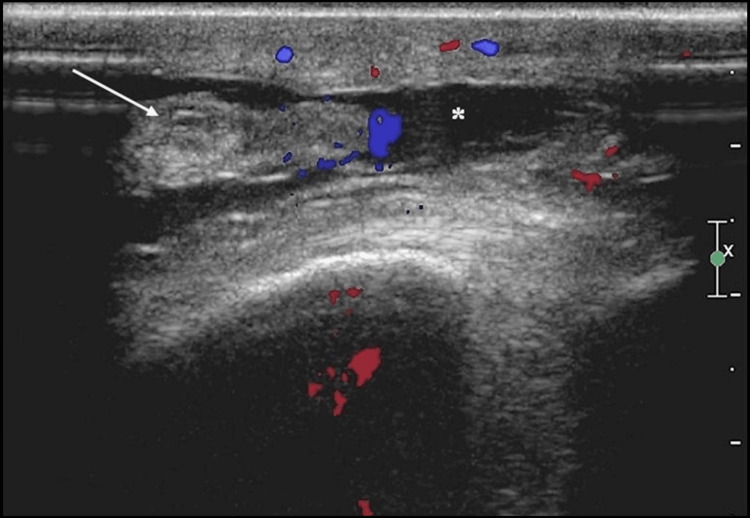
Doppler-ultrasound image of prepatellar soft tissue revealing a well-defined fluid collection (asterisk) within subcutaneous tissue containing some fat lobules (arrow) with no hyperemia.

Subsequent ultrasound examination unveiled a fluid effusion within the deep subcutaneous prepatellar soft tissue (Figure 2).

**Figure 3:**
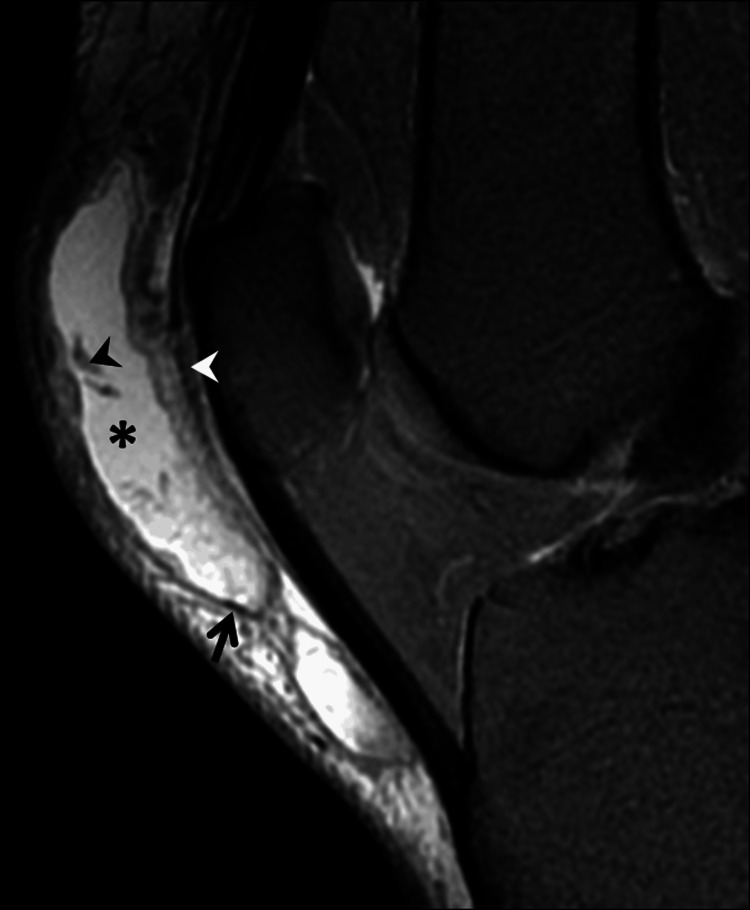
Sagittal view of PD-FS MRI sequence demonstrating a hyperintense prepatellar fluid collection (asterisk) containing hemorrhagic debris (black arrowhead) and featuring a well-defined capsule (black arrow). Note the longitudinal fibers of the rectus femoris tendon extending to the patellar tendon (white arrowhead). PD-FS, proton density fat-saturated; MRI, magnetic resonance imaging

First, a diagnosis of prepatellar bursitis was established based on clinical and imaging findings. MRI was conducted approximately two months post-injury to confirm the diagnosis, which revealed a unilocular well-defined fluid collection. This collection appeared hypointense on T1-weighted images (T1-WI) and hyperintense on proton density fat-saturated (PD-FS)-weighted images, situated directly overlying the patella and patellar tendon (Figure 3).

**Figure 4:**
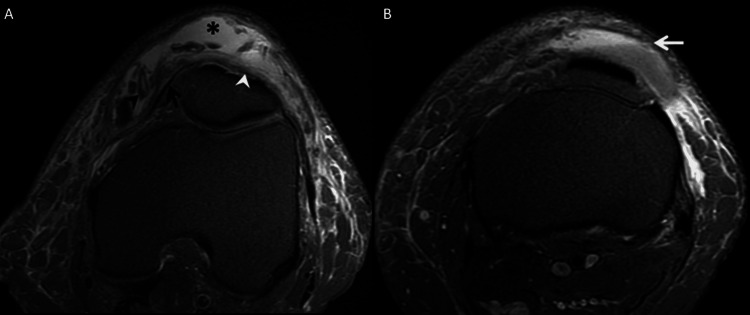
(A) Axial view of PD-FS MRI sequences demonstrates a hyperintense prepatellar fluid collection (asterisk) containing hemorrhagic debris (black arrowhead) and featuring a well-defined capsule (black arrow). The longitudinal fibers of the rectus femoris tendon extend behind the collection (white arrowhead). (B) Axial slice of a DP-FS sequence showing a blood-fluid level (white arrow). PD-FS, proton density fat-saturated; MRI, magnetic resonance imaging

Specifically, the collection was localized within the deep subcutaneous prepatellar fat, anterior to the prepatellar longitudinal fibers of the rectus femoris tendon extending to the patellar tendon and exhibited a fluid-fluid level, indicative of a blood-fluid level, encapsulated by a thin hypointense wall. Additionally, small tissue debris was discernible within the collection, potentially representing remnants of fat lobules, which are suppressed in the FS sequence in association with residual blood debris (Figure 4).

Importantly, no associated intra-articular pathology was identified during the imaging evaluation. No contrast injection was performed, given the absence of a solid component within the mass and the lack of clinical suspicion for malignant and infectious pathology. The relationship of the collection with the fascia aligns with a diagnosis of PMLE. Management was conservative associating knee compression with reducing physical efforts leading to a decrease in the collection and effusion after a non-response to non-steroidal anti-inflammatory drugs.

## Discussion

PMLE ensues from the formation of a prepatellar collection induced by excessive shearing force or repetitive compressive trauma, leading to the separation of the subcutaneous fat tissue from the underlying deep fascia. This disruption of the hypodermic vascular plexus, encompassing vessels and lymphatics, precipitates an accumulation of lymph, blood, and fat lobules within the interfascial plane, resulting in the gradual or rapid development of a heterogeneous collection depending on the involvement of lymphatic or arterial beds. Without intervention, a secondary inflammatory reaction may ensue, organizing around the collection and forming a peripheral fibrous pseudocapsule, impeding effusion resorption and fostering persistent fluid accumulation [2].

Characteristic manifestations of PMLE encompass pain and prepatellar swelling, with physical examination often revealing a soft, fluctuant area of contour deformity. While Morel-Lavallée effusion is classically associated with the hip (36% of cases), thigh (24% of cases), and pelvis (19% of cases), its occurrence at less common sites such as the knee represents a rarity (15% of cases) [2].

Ultrasound and MRI serve as pivotal modalities for confirming the diagnosis of PMLE while also excluding alternative pathologies. The appearance of PMLE is contingent upon factors including the age of the collection, the composition of blood, fat, and lymphatic tissues within it, and the presence of secondary inflammatory reactions [3]. On ultrasound imaging, Morel-Lavallée lesions exhibit compressibility and lack flow on color Doppler. Acute and subacute lesions (less than one month old) typically manifest with a heterogeneous appearance characterized by irregular margins and a lobular shape, while chronic lesions (greater than 18 months old) tend to display homogeneity with smooth margins and a flat or fusiform shape [2].

Mellado and Bencardino introduced a classification system comprising six types of Morel-Lavallée lesions, predicated upon factors including shape, presence or absence of a capsule, overall signal characteristics on T1 (T1-WI) and T2-WI, in addition to the enhancement features discerned through MRI. Type I Morel-Lavallée lesions are designated as seromas, presenting homogeneous hyperintense collections on T2-WI, and homogeneously hypointense on T1-WI, lacking evidence of outer capsule formation. Type II lesions represent subacute hematomas, typically exhibiting homogenous hyperintensity on both T1-WI and T2-WI, with a hemosiderin-laden hypointense capsule. Chronic organizing hematomas, classified as type III lesions, demonstrate hypointensity on T1-WI and heterogeneous hypointensity/isointensity on fluid-sensitive images. Type IV lesions denote closed lacerations, characterized by the absence of a capsule, T1 hypointensity, T2 hyperintensity, and variable enhancement. Type V lesions showcase a small, rounded, pseudonodular appearance with variable T1 and T2 intensity, alongside areas of both internal and peripheral enhancement. Finally, type VI lesions signify superimposed infection, often accompanied by a thick enhancing capsule and occasionally, an associated sinus tract [4].

The differential diagnosis of PMLE primarily involves prepatellar bursitis, with a key distinguishing feature being PMLE's characteristic extension beyond the normal margins of the prepatellar bursa. Typically, these margins are confined to the mid-coronal plane both medially and laterally and to the mid-thigh proximally. However, in our study, we did not observe this typical extension of the collection; Instead, other signs were present guiding the diagnosis. Notably, PMLE size typically remains unchanged on follow-up imaging due to the presence of a fibrous pseudocapsule while a response to steroid injection may be seen in bursitis [2,4]. In certain instances, Morel-Lavallée lesions can present diagnostic challenges, mimicking neoplastic soft tissue lesions such as sarcomas. This consideration is particularly pertinent in cases corresponding to type V and VI Morel-Lavallée lesions, characterized by a heterogeneous appearance accompanied by involvement of surrounding soft tissue abnormalities and chronic symptoms. A key discriminant from a Morel-Lavallée lesion is the presence of areas demonstrating avid homogeneous internal contrast enhancement, a feature not typically observed in Morel-Lavallée lesions, even those of type III classification. This contrast enhancement pattern serves as a crucial distinguishing factor aiding in the differentiation between Morel-Lavallée lesions and neoplastic soft tissue lesions such as sarcomas [2]. Other differential diagnosis including fat necrosis resulted in soft tissue indentation with reduced volume of fat following a direct impact in addition to lymphatic malformations and abscess. In most cases, clinical data may assist in guiding diagnosis along with the specific features of each disease [5].

In Morel-Lavallée lesions regardless of the location, an initial conservative approach is warranted. This typically involves the application of compression bandages, either alone or in conjunction with sclerotherapy. For lesions that persist despite compression, percutaneous drainage may be considered an adjunctive measure [2,6]. Current literature advocates more for conservative approaches. A study focused on knee Morel-Lavallée lesions in football players shows a promising outcome by aspiration of the fluid followed by compression as a minimally invasive approach avoiding surgical drainage with hospitalization necessity and reducing the recovery period [7]. Conversely, chronic lesions necessitate a more proactive therapeutic approach. Initial treatment strategies should involve percutaneous drainage coupled with sclerotherapy. It is imperative to recognize that managing chronic lesions with sclerotherapy may prevent patients from recurrent postoperative hematomas and secondary infections [8]. Surgical approach may be warranted in complicated cases carrying a heightened risk of morbidity and mortality such as perineal lacerations and bone- or muscle-associated lesions [2]. A minimally invasive technique has proved its efficiency with better cosmetic results and fewer wound complications by debridement of the chronic capsule of the Morel-Lavallée collection with an endoscopic resection approach [9].

## Conclusions

In conclusion, the prepatellar localization of Morel-Lavallée injury represents a relatively uncommon occurrence. It is imperative to underscore the significance of recognizing PMLEs, particularly within the context of trauma or contact sports such as football and wrestling, where misinterpretation as bursitis is plausible. Utilization of imaging including ultrasound and MRI in delicate cases serves as a tool facilitating the distinction between these entities, leading to offering therapeutic insights and guiding appropriate management strategies as well as excluding alternative differential diagnoses that may closely mimic PMLE in both radiologic and clinical presentation. Conservative measures predominantly suffice, particularly in acute phases, while surgical intervention is reserved for chronic or complicated cases where conservative management proves to be inefficient.

Thus, a comprehensive understanding of the clinical, radiological, and therapeutic aspects of PMLEs is essential for optimizing patient care and outcomes.
